# Clinical Evaluation of Novel Custom 3D-Printed Meshed-Silicone Orthotics Utilizing Standing Foot Scans and Dynamic Gait Data

**DOI:** 10.1177/11795972251371476

**Published:** 2025-10-15

**Authors:** Joshua Kubach, Mario Pasurka, Julia Lueg, Marcel Betsch

**Affiliations:** 1 Department of Trauma Surgery and Orthopaedics, Friedrich-Alexander-University Erlangen-Nuremberg, University Hospital Erlangen, Germany

**Keywords:** 3D printing, orthotics, biomechanics, leg axis correction, customized insoles

## Abstract

**Background:**

Conventional orthotic insoles demonstrate limited accommodation for individual foot morphology and plantar pressure distribution patterns, resulting in biomechanical inefficiencies and patient discomfort. Computational approaches integrating artificial intelligence with additive manufacturing technologies offer promising solutions for personalized orthotic design. This study investigates the clinical efficacy of AI-driven 3D-printed meshed-silicone orthotics through comprehensive biomechanical assessment.

**Methods:**

A prospective cohort study (n = 21; 8 females, 13 males; age 25.6 ± 3.68 years; BMI 25.48 ± 3.46) evaluated custom orthotics fabricated using machine learning algorithms applied to individual foot and gait data. Pre- and post-intervention assessments included Visual Analog Scale (VAS), Foot Function Index (FFI), Foot Posture Index (FPI), plantar pressure distribution analysis, and 3-dimensional gait analysis over a 4-week period.

**Results:**

FFI scores showed minimal variation (pre-intervention: 13.48 ± 13.14; post-intervention: 14.10 ± 12.96). Significant biomechanical modifications were observed: multi-planar lower extremity alignment correction at hip, knee, and ankle joints. Plantar pressure redistribution demonstrated decreased heel loading with unchanged forefoot pressure distribution, accompanied by significant maximum metatarsal pressure elevation (*P* < .05).

**Conclusions:**

II. AI-integrated 3D-printed meshed-silicone orthotics demonstrated measurable biomechanical improvements including lower extremity alignment optimization and plantar pressure redistribution. These computational design methodologies combined with advanced manufacturing technologies establish a foundation for personalized orthotic interventions in clinical biomechanics applications.

## Highlights

- Individualized novel 3D-printed meshed silicone were obtained using static as well as dynamic data.
- A machine learning algorithm was utilized to obtain optimal pressure distribution to correct leg axis and shift pressure distribution around the forefoot.

## Introduction

Our feet are as unique as fingerprints, yet we confine them in standard shoes and insoles. Traditional shoe insoles often fail to adapt to the specific contours and pressure distribution of each individual’s feet, resulting in discomfort and foot health problems. Insoles may help improve foot health, increase comfort, and relieve pain.^1-3^ Custom orthopedic orthotics can also be used to treat various foot pathologies, such as splayfoot, plantar fasciitis, posterior tibial tendon dysfunction, diabetic foot syndrome and leg length discrepancy.^4-11^

The subjective nature of comfort perception is well-established in the literature; however, research has identified several objective parameters that correlate with perceived shoe comfort.^1,12,13^ These factors include the tactile softness of the shoe interior, thermoregulatory properties (covering both temperature and humidity management), overall flexibility, weight, shock attenuation, impact absorption in the heel region, and the distribution of plantar pressures.^12,14^ Standard orthotics can address various types of foot arch imbalances. The most common deformities are pes planus, which has a prevalence of up to 50%, including up to 30% in the asymptomatic population,^15^ and pes cavus, with a prevalence of up to 15%.^16^, Traditional insole manufacturing is often done in a standardized way that may not consider the individual needs of each patient.

In orthopedic and podiatric applications, insole manufacturing has significantly improved with both subtractive and additive manufacturing techniques. Subtractive manufacturing, traditionally used for making insoles, involves machining material blocks. While this method offers high precision, it leads to material waste, limited material options, and presents challenges in creating internal structures.^17^

In contrast, additive manufacturing, particularly 3D-printing, has emerged as a promising alternative for orthotics production.^6,18,19^ This technique enables the creation of complex shapes, multi-material constructions, and the integration of functional elements within the insole. The layer-by-layer approach allows for customization based on individual foot shapes and specific orthopedic needs. With advances in manufacturing, new and diverse materials can now be^16,20^ used for 3D-printed insoles. To date, Ethylene Vinyl Acetate (EVA) and Polypropylene (PP) have been the most commonly used polymers in shoe insole production.^21^ However, there are multiple limitations regarding traditional materials in 3D printing, especially in terms of flexibility, breathability and durability. To date, no commercially available insole can be individualized to the unique anatomy of every wearer.

Therefore, in this study a novel 3D-printed meshed silicon polymer was used to create a custom, individualized shoe insole. Silicone has excellent properties for use in orthotics. Empirical studies by Shakouri et al have shown that liquid silicone outperforms conventional midsole materials like PP and EVA. This superiority is evident in 2 areas: improved strain energy absorption and more effective plantar pressure distribution. These attributes make silicone a particularly promising material for midsole fabrication in footwear engineering, potentially offering significant advancements in both comfort and functional performance compared to traditional material options.^22,23^

Recent studies have demonstrated the potential of additive manufacturing for creating biomechanically optimized insoles with varying densities and support structures.^8^

The objective of this study is to validate the theoretical basis for personalized 3D-printed silicone insoles with a mesh structure. This will be accomplished by employing a scalable diagnostic and printing approach to examine both subjective and biomechanical properties. The ultimate goal is to establish a robust framework for the mass production of customized orthopedic insoles.

## Materials and Methods

### Participants

This investigation employed a prospective cohort study design conducted over a 4-week intervention period with 21 volunteers to evaluate the clinical effectiveness of custom 3D-printed meshed-silicone orthotics as a pilot study.^24^ Twenty-one healthy individuals were selected based on strict inclusion and exclusion criteria. Eligible participants had to exhibit no significant podiatric pain. Those with diagnosed pronation misalignment or clinically significant foot conditions, such as for example, polyneuropathy or skin lesions, were excluded for this pilot study. All participants were informed about the study, provided written consent, and were given the option to withdraw at any time. The study protocol was approved by the local ethics committee (Number: 23-154-B).

### Datapoints

The following data was recorded: age, weight, height, BMI, shoe size, and type of shoes worn. The Foot Posture Index (FPI) was recorded, it is a validated tool for assessing foot posture, with scores indicating supination, neutral, or pronation.^25^ The Foot Function Index (FFI) measures foot pain and disability across multiple domains.^26^ Callus formation was assessed by nominal scale classification described by,^27^ with each foot zone graded on a standardized scale: light callus (Grade 1), heavy defined callus (Grade 2), concentric keratin plugs (Grade 3) and callus with deeper density changes under the forefoot (Grade 4). Examinations were performed by a trained clinician at baseline and after the intervention period of 4 weeks. The selected 4-week observation timeframe achieves an appropriate equilibrium between ensuring sufficient adaptation duration and preserving safety protocols for initial assessment, aligning with methodological approaches utilized in multiple comparable insole pilot studies.^28-30^ Furthermore, VAS comfort level^31^ was also assessed, ranging from 0 to 10, with higher scores indicating greater comfort. We recorded subjective data focusing on comfort, material quality of the insoles, as well as shoe climate. Subjective comfort, material quality and shoe climate were rated on a 0% to 100% scale, with scores above 75% classified as high and scores above 65% as good.

Participants tracked their step count through native smartphone apps. Furthermore, participants were ask to rate their weekly moderate (Examples: carrying light loads, cycling at a regular pace, doubles tennis, dancing, gardening and intense activity (Examples: heavy lifting, aerobics, fast cycling, running, football activity) and was adapted from.^32^

Objective data was measured using a Gait Laboratory with pedobarographic sensors built into a treadmill (Formetric 4D motion, Diers International GmbH, Schlangenbad, Germany).

### Novel Materials and AI-Based Orthotics Design Proposal

The custom-built shoe insole integrates individual gait data, 2-component silicone rubber and additive manufacturing (3D printing) technology to create customized biomechanical silicone insoles. Two-component silicone rubber was chosen as the material for 3D-printing the custom insole due to its favorable performance characteristics. Thermoset silicone offers excellent dimensional stability and retains its material properties and flexibility across a temperature range from −60°C to +200°C. Additionally, silicones are widely used in medical devices and human implants because of their high biocompatibility.

Silicone rubber provides a water-repellent surface with low moisture uptake, thereby preventing mold or bacterial growth. Combined with silicone’s low compression set of silicone, meaning it resists permanent deformation under sustained load, this makes it an ideal material for orthotics. The silicone rubber was specifically formulated to combine the performance benefits of silicone elastomers with the design and processing advantages of liquid additive manufacturing (3D printing). It has a low viscosity for smooth printing and unique rheological properties for high resolution and accuracy.

The elasticity and recovery of the material optimize energy return, cushioning, and foot stability. While the material properties mentioned above were all studied by Vrije University Amsterdam, Netherlands, only the effects of 3D–printed silicone midsole design on gait biomechanics have been published.^22^ Philippart et al^22^ specifically highlight the positive effects of the elastic material on plantar pressure distribution by increasing the contact area around the midfoot.

A common issue with 3D-printed structures is that they are often made from relatively stiff materials, making the structure inflexible due to the material composition used for 3D-prinitng. To create a 3D-printed structure with compressibility and flexibility while still maintaining structural integrity, a layered honeycomb design with cells of varying thicknesses was developed. Typically, such structures are used in the aerospace industry to provide stiffness, where materials must retain their shape under external forces and in the case of orthotics remain comfortable.^33,34^ While 3D-printed structures for custom foot orthotics have been explored in recent studies, our approach utilizing 3 patents for a unique meshed silicone structure represents an incremental advancement in flexibility and customization.^4,10,35^

**Figure 1.**
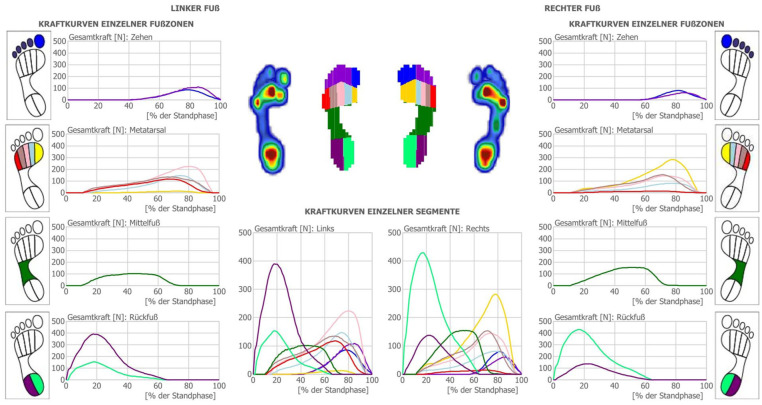
Force diagram for force distribution for the 10 different regions of the foot (medial and lateral rearfoot, midfoot, the 5 metatarsals, hallux and toes 2-5).

**Figure 2.**
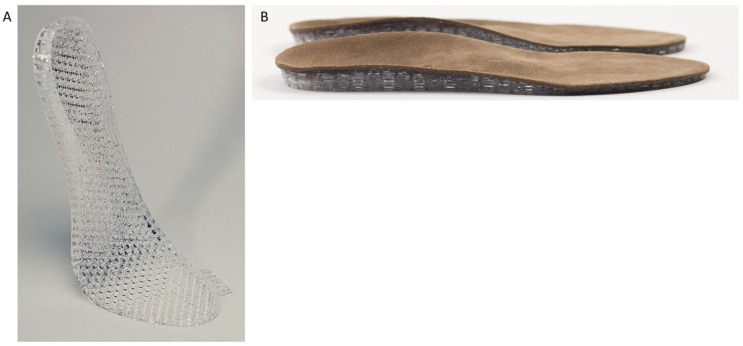
(A) 3D-printed honeycomb meshed insole. (B) Lateral view of the insole featuring a 3D-printed base and a leather upper layer.

Advanced AI and machine learning algorithms were employed to analyze static and dynamic pressure measurements of the human foot, optimizing support and corrective interventions. The process begins by segmenting the pressure image of the foot into 10 distinct regions: medial and lateral rearfoot, midfoot, the 5 metatarsals, hallux, and toes 2 to 5 (Figure 1). To refine the calculations for these foot zones, a dedicated AI project was developed. Using data from force and pressure readings within these zones, machine learning models predict the ideal shore value and orthotic shape, accommodating various foot arch types, including flatfoot, normal, and high-arched feet. These AI models were trained on a dataset comprising over 20 000 measurements from a diverse population, enabling precise analysis and reliable orthotic recommendations. The models utilize advanced neural network architectures capable of learning complex relationships between pressure distributions, foot morphology, and necessary corrections, ensuring the generated orthotics are highly individualized and accurate. Dynamic pressure readings are a critical component of the system, revealing not just pressure distribution but also its variation over time. This temporal data provides insights into the movement of the center of pressure (CoP) and local pressure shifts, helping identify whether additional support is needed in the rearfoot, midfoot, or forefoot regions (Figure 2). Machine learning algorithms analyze this temporal data to detect patterns indicative of biomechanical inefficiencies or abnormalities, further refining the orthotic design. Once the basic parameters, such as arch type and shore value, are calculated, the system determines additional corrective elements. These include medial rearfoot support, medial midfoot support, medial forefoot support, lateral midfoot support, and metatarsal support (pelotte). The AI system evaluates the interplay between these corrective elements to suggest a comprehensive orthotic design tailored to the user’s unique needs.

By integrating AI and machine learning algorithms to analyze both static and dynamic pressure measurements, along with a supervised machine-learning approach that consolidates data from the leg axis, hip, and spine, Sequences’ system enables the generation of measurable and reproducible outcomes. This approach extends beyond traditional foot measurements and the development of individualized, 1-off proposals.

### Gait Analysis

Participants were examined using the Formetric 4D motion gait lab (Diers International GmbH, Schlangenbad, Germany) during both standing and walking. Video gait analyses of the legs from all angles were recorded with 4 high-speed cameras. The synchronous measurement of leg alignment and foot pressure during walking allows for the identification of abnormalities in movement patterns. A standardized protocol was followed in which participants walked for 60 seconds at a speed of 4.5 km/hour. Gait parameters were then recorded over a 10-second interval at a constant speed of 4.5 km/hour. Leg axis geometry was captured via video, with self-reflective markers placed on defined points and tracked by the cameras. Leg alignment was assessed using joint angle measurements: For the hip, anatomical landmarks around the pelvis and femur are tracked to calculate the frontal plane hip angle from the femoral axis. At the knee, femoral axis and tibial axis are analyzed to measure the hip-knee-angle. For the hindfoot, the tibial axis and hindfoot axis at the ankle and subtalar joint assess hindfoot angle.

### Statistical Analysis

The quantitative data of clinical scores and measurement results of gait analysis are presented as mean and standard deviation. The Shapiro-Wilk test was used to analyze data distribution. In cases of normality (Gaussian distribution), the paired t-test was applied; otherwise (without a normal distribution), the Wilcoxon rank-sum test was used. P values of <.05 were regarded as statistically significant. The analysis was conducted with the SPSS statistics program (IBM Deutschland GmbH, Ehningen, Deutschland) for Windows (Microsoft Corporation, Redmond, USA).

## Results

### Demographic Data of Participants

Twenty-one healthy participants (8 females, 13 males) with diverse foot types were recruited from the medial faculty of the local university (Table 1).

**Table 1.** Demographic Data of Participants.

Parameter	Data (n = 21; mean ± SD)
Age (years)	25.6 ± 3.68
Height (cm)	179.2 ± 9.35
Weight (kg)	81.95 ± 12.64
BMI (kg/m^2^)	25.48 ± 3.46
Shoe size	42.24 ± 3.23
Daily step count	8250 ± 1987
Average moderate daily activity (min)	45 ± 32
Average intense daily activity (min)	22 ± 15

### Clinical Data Assessment and Subjective Data Evaluation

#### Foot Posture Index

Ten subjects’ feet were classified as supinated (47.6%), while the other 11 had a neutral foot posture index (52.4%).

#### Callus Formation

**Figure 3.**
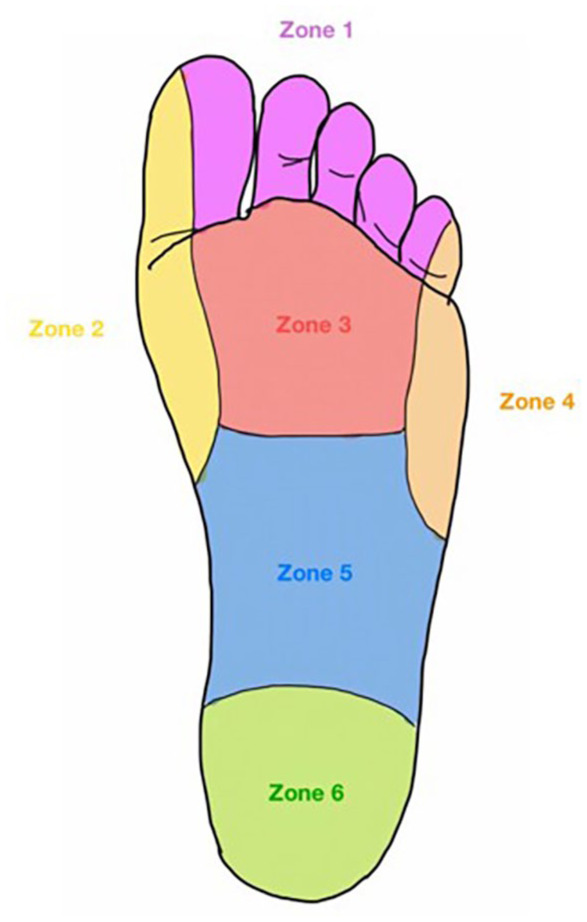
Division of foot zones with callus measurements.

The rating of the callus formation was divided for each zone (Figure 3) according to the following criteria: light callus (Grade 1), heavy defined callus (Grade 2), concentric keratin plugs (Grade 3), and callus with deeper density changes under the forefoot (Grade 4). The majority of the volunteers reported a significant reduction in callus formation with insole wear during the study period across all foot zones (from −3.3% to 24.4%) in both feet. (Table 2).

**Table 2.** Changes of callus formation during the study period.

Zone	Mean before insoles (*t*0)	Mean after insoles (*t*1)	*P*-value (*t*0 vs. *t*1)
Zone 1 left	1.24 ± 0.83	0.95 ± 0.59	.028*
Zone 2 left	2.29 ± 0.78	2.05 ± 0.67	.002*
Zone 3 left	1.48 ± 0.93	1.33 ± 0.97	.189
Zone 4 left	2.10 ± 0.77	1.86 ± 0.73	.067
Zone 5 left	0.71 ± 0.72	0.81 ± 0.81	.165
Zone 6 left	2.19 ± 0.60	2.05 ± 0.67	.093
Zone 1 right	1.24 ± 0.70	0.90 ± 0.54	.002*
Zone 2 right	2.38 ± 0.80	2.00 ± 0.77	.001*
Zone 3 right	1.43 ± 0.87	1.24 ± 0.89	.081
Zone 4 right	2.14 ± 0.73	1.86 ± 0.65	.028*
Zone 5 right	0.67 ± 0.73	0.67 ± 0.80	.5
Zone 6 right	2.00 ± 0.71	1.67 ± 0.66	.008*

*p<0.05.

### Foot Function Index

The mean FFI^36^ before insole use was 13.14 (SD ± 13.57) and after a 4-week period of insole wear it was 12.95 (SD ± 12.89; Table 3). No significant changes for the overall score, nor for the subscales were found.

**Table 3.** Results Comparing Foot Function Index Before Insoles and With Insoles.

FFI	Mean before insoles (*t*0)	Mean with insoles (*t*1)	*P* (*t*0 vs. *t*1)
Pain subscale (0-90)	10.57 ± 10.73	9.95 ± 10.00	.332
Disability subscale(0-90)	1.00 ± 3.30	1.52 ± 3.66	.154
Activity limitation subscale (0-50)	1.57 ± 2.66	1.62 ± 2.82	.288
Scoring (0-230)	13.14 ± 13.57	12.95 ± 12.89	.453

### Subjective Data Evaluation

**Figure 4.**
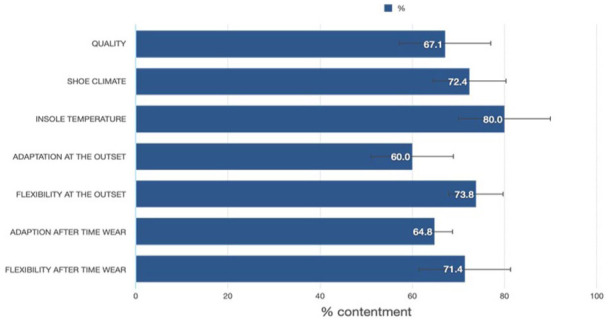
Ranking of insole quality (0%-100%).

All volunteers reported high subjective comfort levels and overall good quality of the insoles (Figure 4).

### Leg Axis Analysis

The orthotics led to an adjustment in leg alignment at the level of the hip, knee, and ankle joints. The frontal plane hip angle decreased significantly with insole wear to −4.57° on the left (*P* = .006) and −4.67° on the right (*P* = .005). The hip-knee-angle decreased significantly with insole wear to 0.71° on the left (*P* = .002) and 2.71° on the right (*P* < .001). The hindfoot angle decreased significantly with insole wear to −1.19° on the left (*P* = .008) and 0.14° on the right (*P* = .011; Table 4).

**Table 4.** Results of Leg Axis Analysis (*t*0: Without Insoles, *t*1: With Insoles).

	Without Insoles (*t*0)	With Insoles (*t*1)	*P* (*t*0 vs.*t*1)
Left hip (°)	−6.33 ± 4.11	−4.57 ± 0.74	.006
Right hip (°)	−7.33 ± 4.27	−4.67 ± 3.31	.005
Left knee (°)	−2.33 ± 5.81	0.71 ± 6.11	.002
Right knee (°)	−2.19 ± 6.81	2.71 ± 6.33	<.001
Left ankle (°)	−3.19 ± 3.44	−1.19 ± 4.04	.008
Right ankle (°)	−2.90 ± 4.80	0.14 ± 4.62	.011

### Gait Parameters

**Figure 5.**
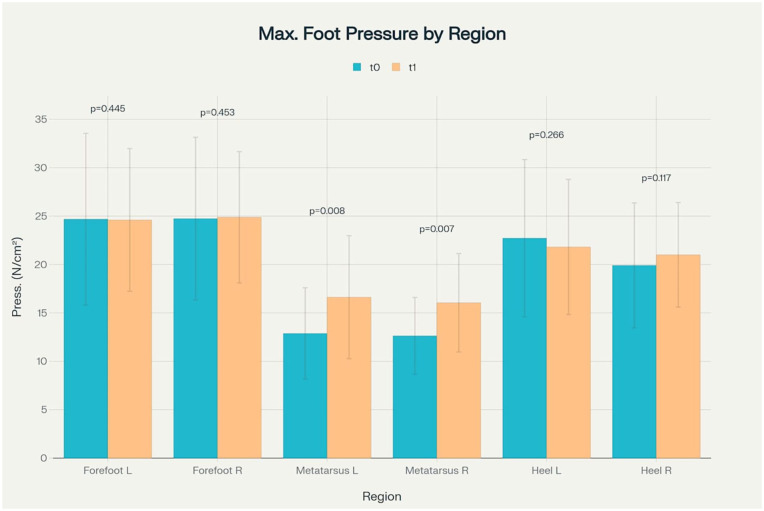
Comparison of maximum plantar pressure measurements at baseline (*t*0) and follow-up (*t*1) across different foot regions, showing means with standard deviations and statistical significance values.

The 3D-printed orthotics did not significantly affect the respective gait phases (Table 5). The custom-built shoe insoles redistributed foot pressure during walking. Areas around the heel experienced a slight, non-significant decrease in plantar pressure, while the forefoot maximum pressure with insole wear did not change during normal walking (measurements at 4.5 m/s). Due to the added support under the foot, we found a significant increase in maximum metatarsal pressure with insole wear (Figure 5).

**Table 5.** Gait Phases Recorded At Walking Speed 4.5 km/hour (*t*0: Without Insoles, *t*1: With Insoles).

Gait phase	Without Insoles (*t*0)	With Insoles (*t*1)	*P* (*t*0 vs. *t*1)
Loading response phase left (%)	12.20 ± 1.23	12.49 ± 1.16	.118
Stance phase left (%)	64.89 ± 1.25	64.79 ± 1.32	.355
Single support left (%)	38.37 ± 1.40	37.69 ± 1.11	**.021**
pre-swing phase left (%)	14.91 ± 1.40	15.02 ± 1.22	.341
Swing phase left (%)	35.11 ± 1.25	35.21 ± 1.32	.355
Loading response phase right (%)	14.88 ± 1.37	14.92 ± 1.36	0.436
Stance phase right (%)	64.73 ± 1.69	65.19 ± 1.27	0.78
Single support right (%)	35.01 ± 1.29	35.15 ± 1.35	0.314
pre-swing phase right (%)	14.84 ± 1.43	15.09 ± 1.21	0.132
Swing phase right (%)	35.27 ± 1.69	34.81 ± 1.27	0.078
bipedal phase (%)	14.83 ± 1.31	15.07 ± 1.18	0.149

## Discussion

The utilization of 3-dimensional foot scanning technology and dynamic pedobarographic measurements presents significant potential in orthotics product design. Historically, footwear and shoe insole production primarily relied on 2-dimensional foot measurements, such as foot length and width. However, this technology accounts for the dynamic changes in foot morphology and plantar pressure throughout the gait cycle and how these changes are influenced by different types of footwear. The advantage of customized and 3D-printed insoles should also provide high precision and fit, as well as a reduction in localized foot pressure distribution, which is a significant issue for patients with various foot deformities.^19^

Until now different studies have compared additive and subtractive manufacturing methods with an increasing number of studies utilizing individualized insoles due to advancements in machine and deep-learning methods as well as advances in 3D printing technology.^5,7,8,19,21,37,38^ In contrast to these studies, our evaluation focused on proving the concept of a novel manufacturing process using 3D printable meshed silicone insoles tailored to each individual foot, utilizing pedobarographic foot scan technology (Diers International GmbH, Schlangenbad, Germany). While 3D printing of silicone orthotics has been employed in other medical devices, its application in insole manufacturing has so far been to biomechanical studies.^21,22^ The integration of machine learning for orthotics design proposal extends beyond the initial analysis. The system continuously learns from new data, improving its predictive accuracy and the quality of its recommendations. For instance, data from leg axis, hip, and spinal analysis are already utilized in supervised machine learning to recommend appropriate adjustments in cases of leg length discrepancies. By incorporating feedback from clinical outcomes and user satisfaction, the models adapt to emerging trends and refine their decision-making processes. This AI-driven approach not only ensures high precision and consistency but also significantly reduces the time required for orthotic customization, making advanced foot care more accessible and efficient.

In the present study, we clinically and biomechanically evaluated novel orthotics utilizing a gait laboratory. We demonstrated high wearing comfort while maintaining the durability and breathability of the aforementioned insoles. We also observed a shift in plantar pressure to the midfoot, with a redistribution of maximum plantar pressure from the heel to the midfoot. These benefits were achieved despite no significant differences in pain scores or limitations in activity scores, as described in the Foot Function Index^36^ before and after the intervention in a healthy population.

As described previously, a redistribution of plantar pressure to the midfoot can have beneficial mechanical effects on the function of the midfoot as a fulcrum.^20,39^ The foot’s arch-area can function as a lever arm against the ground reaction force, allowing the foot to efficiently transfer forces and propel the body forward during walking. Comparable results were found by Khodaei et al, who investigated CAD-CAM insoles in flexible flatfoot deformities,^7^ and by Xu et al, who studied 3D-printed insoles on plantar biomechanics in patients with plantar fasciitis.^8^ In our study, this potential benefit was observed while forefoot pressure was maintained. These findings support the idea of a shift in plantar pressure and contact area to the midfoot while reducing pressure in the heel area. It also suggests a potential benefit for patients with forefoot pain, which needs to be further researched.

We also found adjustments in leg alignment at the hip, knee, and ankle levels while the subjects were wearing orthotics. Previous research suggests that altering joint angles with orthotics can be beneficial for patients with conditions such as osteoarthritis of the hip and knee,^40,41^ as well as for patients with pelvic obliquity.^42,43^ The adjustment in leg alignment caused by the orthotics did not lead to an increase in pain or discomfort in our subjects, while maintaining equal stride length, peak pressures, and spinal movement.

The individualized orthotics seem to optimize gait symmetry during both walks. This is supported by research presented by Menez et al, who studied patients with mild leg length discrepancies and subclinical leg axis deformities, finding a significant supportive effect of insoles even in patients with leg length discrepancies under 1 cm.^44^ Leg length discrepancy is described in literature as affecting up to 70% of the population,^43^ with studies investigating its impact on lower back pain,^45^ osteoarthritis of the hip and knee,^40^ running economy and associated injuries.^46^

This further suggests that individually printed insoles may assist in correcting smaller deformities through a manufacturing process that focuses on individualization, from the initial design to the final 3D-printed insoles. Until now, mass-insole manufacturing primarily used easily 3D-printable materials, such as polylactic acid or acrylonitrile butadiene styrene.^35^ However, both materials lack the mechanical properties of the meshed silicon recently described and utilized in our orthotics.^22^ Preliminary results show excellent wear and shock absorption, with an apparent shift in pressure distribution from the heel to the midfoot, suggesting a benefit in walking economy. Additionally, the individualization through foot scans and adaption of the silicone mesh led to an adjustment in the leg axis while maintaining comfort, mobility, and pain scores.

This combination suggests a clear benefit of the material in optimizing individualized manufacturing. Although the study period of 4 weeks is rather short, it was previously shown that mechanical and neurophysiological adaptations take place within a few days.^47,48^ For initial proof of concept, it is general practice to observe effects during the first weeks up to a month to balance a practical time period with a period long enough to allow adaptive changes to occur. In comparison to clinical practice and other literature different observation periods from under 1 week to many months are practiced and published. This timeframe balances practicality with a duration long enough to allow for adaptive changes.

Following this successful pilot study, future research should encompass participants with diverse foot morphologies and medical conditions, to evaluate orthotic efficacy across varied presentations. Given the exclusion of individuals with pedal pathologies and elderly participants, subsequent controlled trials should target a broader spectrum of populations to assess the therapeutic effectiveness and safety of 3D-printed meshed-silicone orthotics in managing foot disorders in comparison to standard care. For broader use in a healthy population customers would be able to individually adapt the insole to their preferences and intended uses while balancing optimal biomechanical efficiency.

This study has some limitations that need to be addressed. One limitation of our study is the challenge of defining participants as asymptomatic, given that they were only assessed through a questionnaire and a clinical examination. This approach does not entirely rule out clinically in apparent foot posture pathologies with absolute certainty. Due to the nature of our exploratory clinical study, we did not include a control group or blinding. The lack of blinding and a control group, such as participants using standard commercially available insoles, limits our ability to attribute observed effects solely to the novel orthotic design. Furthermore, as participants were exclusively recruited from a medical faculty, our sample may not be fully representative of the general population. Especially due to high activity levels mainly supinated participants. Future studies should aim to include larger and more diverse cohorts to enhance external validity.

Additionally, since the prototype is still under development, some design features will likely change before the product reaches the market. The 4-week duration of the study prioritizes rapid development but may not be sufficient to capture long-term adaptations, material durability, or delayed adverse effects such as discomfort, arch fatigue, or overuse injuries. Extended follow-up periods are recommended in future studies to thoroughly evaluate these aspects. Due to the study design leg length was not measured. Regarding ankle-to-knee and knee-to-hip lengths we hypothesize they were balanced by different pressure distributions observed in foot pressure measurements, but cannot be proven without a specific measurement and control group. Further research is needed to explore these specific observations. The AI training datasets are proprietary and cannot be disclosed due to commercial confidentiality requirements. This limits our ability to assess dataset representativeness and potential population bias, consistent with common practices in commercial medical AI applications. The machine learning training datasets are proprietary and cannot be disclosed due to commercial confidentiality requirements. This limits our ability to assess dataset representativeness and potential population bias, consistent with common practices in commercial medical AI applications.

## Conclusion

This study demonstrates the effectiveness of a novel, highly innovative 3D-printed insole. The findings indicate that these insoles not only provide high wearing comfort but also possess the potential to redistribute foot pressure effectively. Furthermore, they show promise in correcting leg alignment. These results highlight the advantages of utilizing advanced 3D printing technology and machine learning algorithms in the development of customized orthotic solutions, paving the way for future research and broader clinical applications in the management of various foot and leg conditions.
